# Design of a SoC-Based Highly Integrated RF Transceiver Module

**DOI:** 10.3390/s26134173

**Published:** 2026-07-02

**Authors:** Jianxi Wu, Hao Zhou, Linfeng Shang, Yawei Shao, Kan Wang

**Affiliations:** Nanjing Research Institute of Electronics Technology, Nanjing 210039, China; wjxkbdx@163.com (J.W.); pompom2026@163.com (L.S.); 225132131561@njust.edu.cn (Y.S.); 15910937016@163.com (K.W.)

**Keywords:** application-specific integrated circuit (ASIC), RF transceiver, universality, miniaturization, low power consumption

## Abstract

To address the issues of high customization, long development cycles, and excessive power/volume in radio frequency (RF) transceiver modules for Synthetic Aperture Radar (SAR) and radar systems, this paper presents an ultra-compact universal RF transceiver module design based on a full application-specific integrated circuit (ASIC) architecture. Centered on a wideband RF System-on-chip (SoC) and a reconfigurable digital SoC, the module integrates the complete RF transceiver chain—including filtering, amplification, mixing, Analog-to-Digital/Digital-to-Analog Converter (ADC/DAC) conversion, digital preprocessing, and high-speed data transmission. Test results demonstrate that the 8-channel module achieves a 53.1% area reduction and 55.1% lower power consumption (only 40.9 W) compared with conventional architectures, while all key RF specifications meet system requirements. The proposed solution improves upon existing limitations in high integration, low power, and generality, offering a low-cost, rapid-development technical route for transceiver modules in radar and communication applications.

## 1. Introduction

In recent decades, radar technology has undergone profound transformations, driven by the relentless demand for higher resolution, greater flexibility, and enhanced situational awareness [1,2,3]. Among various radar systems, SAR has emerged as one of the most significant advancements, delivering all-weather, day-and-night imaging capabilities with meter- to sub-meter-level resolution [4,5,6,7]. Today, spaceborne, airborne, and unmanned platforms carry sophisticated SAR payloads that enable military reconnaissance, precision agriculture, disaster monitoring, and scientific remote sensing [5,6]. Multimode SAR systems can switch between stripmap, spotlight, scan-SAR, and interferometric modes, providing three-dimensional topographic mapping and coherent change detection [8,9,10]. Furthermore, bistatic and multi-static SAR configurations, which separate transmitters and receivers, introduce unparalleled advantages such as enhanced anti-interference ability, flexible viewing geometry, and access to non-backscatter target information, greatly expanding the operational envelope of modern radar [3].

These remarkable advances, however, are not achieved in isolation. They critically depend on the progress of the underlying hardware, particularly the digital transceiver modules used in phased-array antennas [11,12]. Contemporary phased-array radars, including those for SAR, are evolving toward fully digital beamforming architectures, in which each radiating element or subarray is driven by an independent Transmit/Receive (T/R) channel [13]. This evolution places stringent requirements on the RF transceiver module: it must handle wide instantaneous bandwidths (often exceeding 1 GHz), support multiple frequency bands, ensure excellent amplitude and phase consistency across channels, and simultaneously reduce size, weight, power, and cost [14]. The digital transceiver module thus becomes the cornerstone that determines the ultimate performance of the entire sensor system [14,15].

In a modern digital array, the transceiver module performs not only signal up/down-conversion, filtering and amplification, but also high-speed data conversion and front-end digital signal preprocessing [16,17]. The increasing scale of arrays—from a few dozen channels to hundreds or even thousands—multiplies the challenges: power consumption, thermal dissipation, and interconnect complexity become dominant design constraints [18]. Traditional architectures, which rely on discrete RF front-ends and intermediate frequency (IF) sampling chains built with separate amplifiers, mixers, filters, data converters, and Field Programmable Gate Arrays (FPGAs), are plagued by high customization, long development cycles, excessive volume, and unacceptable power levels for large arrays [19,20,21]. Each frequency band requires a dedicated RF chain design; integration with digital processing boards is cumbersome; and electromagnetic compatibility issues between high-speed digital and sensitive analog circuits severely limit achievable performance density [11,12].

To overcome these bottlenecks, a paradigm shift towards highly integrated, universal hardware platforms is necessary [22,23,24,25,26,27]. Recent progress in ASICs and SoC technologies has paved the way for such a transition. Wideband RF SoCs now integrate complete up/down-conversion chains covering multiple octaves, replacing entire racks of discrete components. Meanwhile, reconfigurable digital SoCs embed ADCs, DACs, Digital down converters/Digital up converters (DDC/DUC), and high-speed serial interfaces on a single silicon die, drastically reducing board-level complexity. By jointly designing these advanced chips in a synergistic manner, it becomes possible to realize an ASIC-centered RF transceiver module that is not only ultra-compact and low-power but also inherently universal, capable of operating across multiple bands with minimal hardware modification.

Against this backdrop, this paper presents a novel universal RF transceiver module design based on an ASIC-centered architecture, targeting the practical pain points of existing solutions for SAR and multifunction radar systems [25,28,29,30]. The module centers on a custom wideband RF SoC and a highly integrated digital SoC, embodying the entire RF transceiver chain from antenna interface to fiber-optic data output [26]. It achieves dramatic improvements in size, power, and development efficiency. The following sections detail the key technologies, simulation analyses, and experimental verification that underpin this design. The proposed approach offers a low-cost, rapid-development technical route for next-generation digital phased-array and SAR transceivers.

## 2. Design of Key Technologies

### 2.1. ASIC-Centered RF Transceiver Architecture

**Figure 1:**
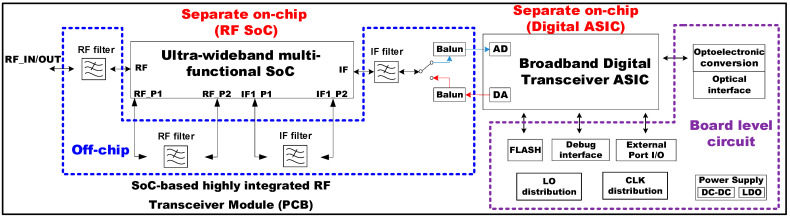
Architecture of the RF transceiver module. CLK: clock; I/O: input/output; DC-DC: direct current to direct current; LDO: low dropout regulator.

The architectural concept of the proposed RF transceiver module is shown in Figure 1. Departing from conventional discrete implementations, the design is built around two core ASICs: an ultra-wideband RF transceiver SoC and a reconfigurable digital processing SoC. The RF SoC requires off-chip filters for filtering and interference suppression. Off-chip baluns are adopted for interconnection between the RF SoC and the Digital ASIC. Supporting circuitry (board level circuit)—including clock generation and distribution, local oscillator (LO) synthesis, optoelectronic conversion modules, power regulation, and control interfaces—is carefully laid out around these chips to form a complete, self-contained transceiver.

Conventional RF transceiver modules use a discrete RF front-end and IF sampling architecture. The RF front-end must be customized for different bands with many components and low integration, making co-integration with IF sampling hard and leading to long cycles, high cost, and large size. IF sampling built with discrete ADCs, DACs, and FPGAs features high power consumption, complex routing, and heavy development efforts.

To address these issues, this paper proposes an ASIC-based architecture combining a wideband RF SoC and a single-chip digital SoC. The ultra-wideband RF SoC replaces discrete RF front-ends, greatly reducing size and power consumption and enabling analog–digital co-integration. The universal architecture also significantly lowers development costs. The highly integrated digital SoC implements IF sampling, reducing components and Printed Circuit Board (PCB) footprint through on-chip integration. On-chip interconnection suppresses electromagnetic interference and crosstalk, while eliminating peripheral auxiliary circuits, further cutting system power consumption and enabling lightweight applications.

The digital SoC operates in a working mode with frequent logic state transitions, which inevitably generates substantial harmonic spurious signals derived from digital clock signals. When realizing high-density co-board integration together with the RF SoC, such spurious digital interference components are highly prone to couple into the adjacent radio frequency signal links. Moreover, some of these digital spurious signals occupy relatively low frequency bands, making them difficult to be effectively eliminated through conventional filtering means.

**Figure 2:**
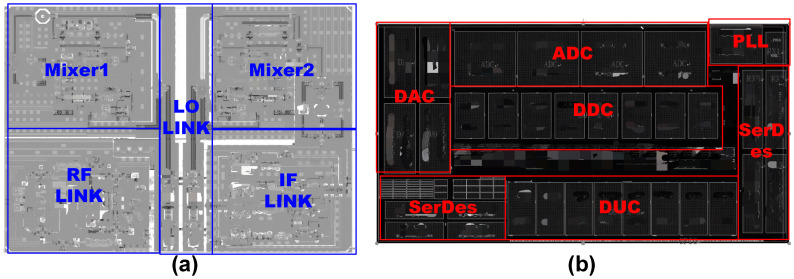
Layout photo of the (**a**) RF SoC and (**b**) digital ASIC.

Figure 2 shows the layout of the ultra-wideband multi-functional SoC (RF SoC) and broadband digital transceiver ASIC (Digital ASIC). The RF SoC integrates dual RF amplifiers, two-stage mixers, IF amplification and filtering chains, and local oscillator distribution chains. The Digital ASIC incorporates functional modules including ADC/DAC, DDC/DUC, phase-locked loops (PLL), and Serializer/Deserializer (Serdes) interfaces.

In this structural design scheme, the printed circuit board is fabricated by adopting 24-layer high-performance FR4 dielectric material, and reasonable partition layout is implemented for digital circuits and analog radio frequency circuits respectively. In terms of power supply distribution and signal wiring routing, thorough layered isolation design is adopted to completely separate digital domains and radio frequency domains. All digital signal lines are laid out with standardized 50 ohm impedance matching routing design, which can effectively suppress the antenna effect induced by digital spurious noises caused by mismatched terminal impedance and incomplete reference ground planes. Considering the large number of interconnection traces between digital and analog functional areas, a unified common ground design is adopted in this work. Sufficient auxiliary grounding vias and isolation grounding vias are densely arranged around all types of signal lines, so that digital signals and radio frequency signals can form independent nearby current return paths respectively. This optimized layout strategy can further suppress the crosstalk interference caused by digital spurious signals and greatly improve the overall electromagnetic compatibility performance of the integrated system.

### 2.2. RF Circuit Design

**Figure 3:**
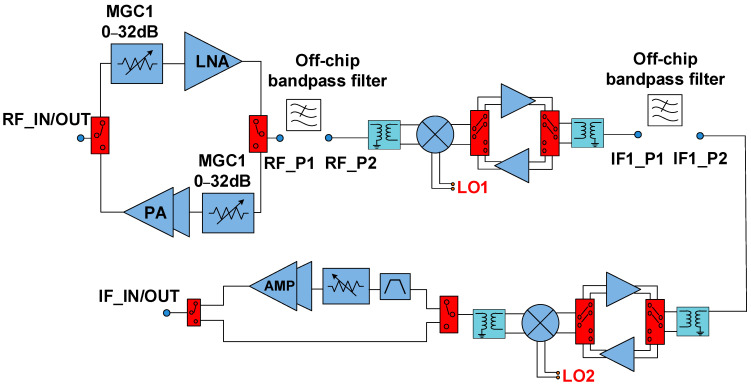
Architecture of the RF transceiver circuit. LO1: local oscillator 1; LO2: local oscillator 2; PA: power amplifier; AMP: amplifier; LNA: low noise amplifier.

The architecture of the designed RF transceiver link is shown in Figure 3. The frequency conversion chip adopted herein is an ultra-wideband integrated multifunctional mixing chip, which is capable of satisfying application requirements. This chip is internally integrated with low-noise amplifier and driver amplifier modules, as well as primary and secondary frequency-conversion functional modules.

An external radio frequency filter interface is reserved between the low-noise amplifier/driver amplifier module and the primary frequency conversion module, which corresponds to the chip pins of RF_P1 and RF_P2. Meanwhile, another external first intermediate-frequency filter interface is arranged between the primary and secondary frequency conversion modules, matching the pin definitions of IF1_P1 and IF1_P2.

The first stage of the entire signal link is composed of a radio frequency filter bank. Users can flexibly select suitable filter devices according to the actual operating frequency bands of practical engineering projects. This filter bank supports various packaging forms such as Low Temperature Co-fired Ceramic (LTCC) and Micro-Electro-Mechanical Systems (MEMS) devices, and can fulfill filtering demands across the full frequency range. In addition, it can be configured into direct-through mode as required by practical applications, which effectively reduces insertion loss at the link input terminal and further optimizes core system performance, including noise figure, overall link gain and output power.

The second stage of the whole system adopts the aforementioned ultra-wideband multifunctional mixing chip. The externally connected radio frequency filter can adopt the same model as the front-stage filter to further suppress out-of-band interference signals and unwanted harmonic components. The external first intermediate-frequency filter can also be selected freely according to actual project demands to adapt to diverse intermediate-frequency configuration schemes.

The third stage is constructed using intermediate frequency filter banks. Given the relatively low operating frequency of intermediate frequency signals, this part mainly adopts LTCC filters and surface acoustic wave (SAW) filters. Relevant devices can be reasonably selected in accordance with the specified intermediate frequency and signal bandwidth of specific application scenarios.

The fourth stage consists of matching circuits for ADC and DAC, together with embedded ADC and DAC intellectual-property core units integrated inside the self-designed wideband digital transceiving ASIC chip.

### 2.3. Digital Circuit Design

**Figure 4:**
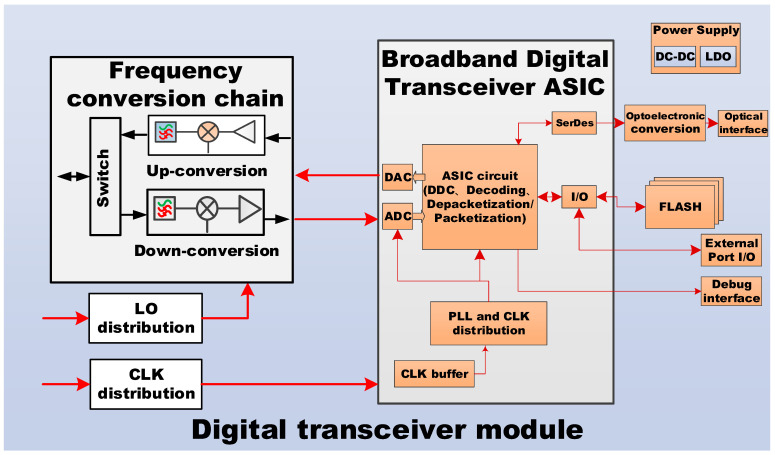
Block diagram of the digital circuit architecture. SerDes: serializer/deserializer; CLK: clock; I/O: input/output.

The block diagram of the digital circuit architecture is shown in Figure 4. The custom-designed digital ASIC chip integrates a full set of functional modules, including ADC, DAC, DDC, DUC, data packing and unpacking unit, clock manager, as well as control and timing parsing modules. When cooperating with other peripheral auxiliary circuits deployed on the digital transceiving module board, it can realize the full-function digital link covering the whole process from signal acquisition and signal generation to optical fiber data transceiving.

The digital transceiving module is capable of converting the externally accessed single power supply voltage into multiple sets of operating voltages required by various internal functional devices, and meanwhile implements effective filtering processing for all power branches so as to maintain stable and reliable power supply throughout the whole system. The digital clock harmonic spurious noises generated by the digital SoC are prone to couple into analog signal links via the internal power distribution network. To alleviate such adverse crosstalk interference, this design adopts a dedicated power isolation and filtering architecture. Starting from the power input terminal, independent LC filter circuits are deployed to separate power supplies for radio frequency circuits and digital circuits, respectively, to strengthen power supply noise suppression. In the subsequent power distribution stage, direct current to direct current (DC-DC) converters and low-dropout linear regulators are adopted in multi-stage cascaded connection, which can greatly suppress the transmission of digital-domain spurious noises to radio frequency links through power lines.

When the system operates in the transmitting mode, the internal clock management circuit receives external clock signals transmitted from the antenna array, and further generates conversion clocks and synchronous clocks that meet the overall system timing requirements inside the circuit board. According to the control commands issued by the control unit and the received symbol data, the digital ASIC chip directly generates radio frequency excitation signals through the built-in DAC modules. The generated signals are then processed via filtering and power amplification before being transmitted to the rear radio-frequency front-end circuit units.

In the receiving working mode, target echo signals captured by the antenna undergo a series of analog signal conditioning processes inside the radio frequency ASIC chip, including low-noise amplification, frequency mixing, digitally controlled attenuation, and bandpass filtering. Afterwards, the processed analog radio frequency signals are directly sampled and digitized by the integrated ADC modules inside the digital ASIC chip, and then transmitted to the digital preprocessing unit. Finally, the formatted digital I/Q signals are packed and converted into optical signals for stable transmission to the back-end signal processing platform.

This wideband digital transceiving ASIC chip integrates eight channels of high-sampling-rate DACs, eight channels of high-sampling-rate ADCs, configurable digital down-conversion logic units, command parsing and decoding units, data packing and unpacking modules, high-speed serial interfaces as well as phase-locked loop modules. By matching with external functional units such as memory chips, photoelectric conversion circuits, power management modules and clock driving distribution links, a complete and integrated digital transceiving module is constructed. Benefiting from the high integration and dedicated customized design of this wideband digital transceiving ASIC, on-chip clock distribution networks and internal power supply resources can be shared by embedded chip IP cores such as ADC, DAC and core digital logic units, which effectively reduces chip layout area and overall power consumption. In addition, all signal interaction and data transmission between ADC, DAC and internal logic circuits are completed inside the chip, featuring extremely short wiring paths and negligible signal attenuation. This design method further cuts down the overall equipment size and reduces the system power consumption remarkably.

### 2.4. Local Oscillator Drive Circuit Design

**Figure 5:**
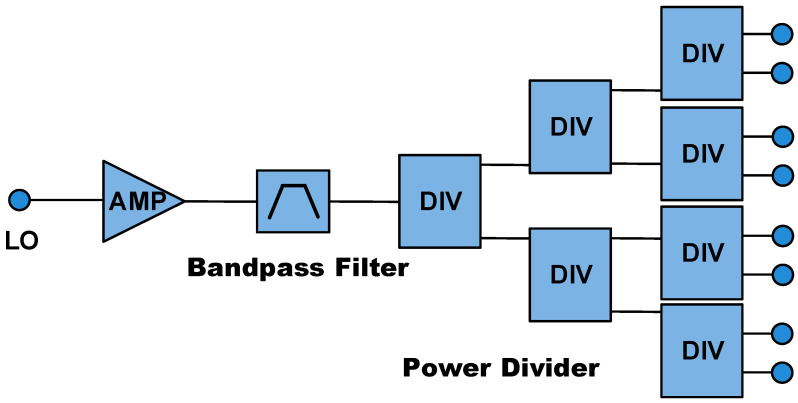
Block diagram of the local oscillator distribution circuit. DIV: divider; AMP: amplifier; LO: local oscillator signal.

The local oscillator driving and power dividing circuit is shown in Figure 5. To reduce the output power requirement of the local oscillator source and simplify the design complexity of the front-end LO circuit, the first stage of the LO link adopts a local oscillator driving amplifier. Since the ultra-wideband integrated multifunctional mixing chip embeds a quadruple frequency multiplier circuit, only low-frequency and quarter-frequency signals need to be input to the primary and secondary local oscillators. Therefore, this driving amplifier adopts the universal PQ4 package. A variety of driving amplifier chips covering segmented quarter-frequency bands of LO signals are commercially available, which can be flexibly selected according to the frequency band and intermediate frequency requirements of practical projects.

The second stage of the link is a bandpass filter, which is used to suppress high-order harmonic components of the local oscillator signal output from the driving amplifier, in order to improve the harmonic suppression performance of radio frequency signals.

The third stage of the link is a power divider network constructed with general LTCC two-way power divider packages. Similarly, numerous two-way power dividers with this package that cover segmented quarter-frequency bands of LO signals can be easily selected to meet various project demands. The proposed LO driving circuit can amplify the input signal from 0 dBm to 17 dBm. The subsequent power dividing link adopts universal LTCC power dividers, ensuring that the divided LO power remains above 2 dBm in multi-band applications, which fully satisfies the application requirements of radio frequency SoC chips.

## 3. Simulation Analysis

### 3.1. Simulation Results of the Transmitting Signal Link

**Figure 6:**
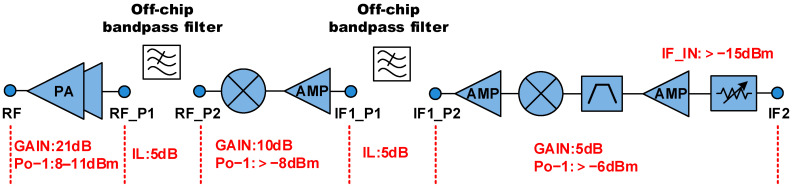
Simulation results of transmission chain performance. PA: Power Amplifier; AMP: Amplifier; RF: Radio frequency; IF: Intermediate frequency.

**Figure 7:**
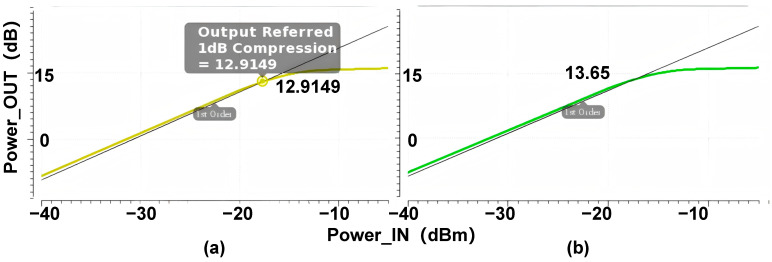
Simulation results of the transmitter output P-1dB at (**a**) 1 GHz and (**b**) 6 GHz.

In order to realize the maximum effective detection range of the radar system and effectively mitigate the adverse influence of gain fluctuation caused by high and low temperature ambient conditions to a certain extent, it is a common engineering design practice to configure the entire transmitting link to operate under a saturated working state in practical circuit design. The relevant simulation analysis results are intuitively displayed in Figure 6. The overall gain value of the complete transmitting link designed in this research is verified to be 26 dB through systematic simulation calculation. Among all functional devices arranged inside the whole signal transmission link, the power amplifier serving as the final-stage functional component is the earliest active device to enter the power saturation operating region, and its stable saturated output power ranges from 8 dBm to 11 dBm accordingly. On this basis, it can be concluded that the intermediate frequency input power injected into the radio frequency SoC needs to be maintained higher than −15 dBm, so as to reliably guarantee that the whole transmitting link can steadily keep working in the expected saturated operating condition. In this system scheme, the second intermediate frequency signal with a fixed frequency of 650 MHz is generated and output by the self-developed wideband digital transceiving ASIC chip, and the full-scale output power of this intermediate frequency signal can reach more than 0 dBm. Such sufficient signal output power can meet the power threshold requirement and guarantee stable saturated operation of the entire transmitting link in actual working scenarios. Figure 7 presents the simulation results of the transmitter output P-1dB.

### 3.2. Simulation Results of the Receiving Signal Link

When the radar system switches to the signal receiving working mode, the entire receiving signal link is required to operate stably within the linear working region strictly. Once the circuit deviates from the ideal linear operating range, unwanted harmonic spurious components together with the truncation effect generated inside analog-to-digital conversion devices will introduce prominent strong spurious interference signals in the subsequent digital signal processing domain, which will seriously hinder the radar system in accurately extracting and effectively identifying valid target echo information. The input 1 dB compression point serves as a core and direct technical indicator to characterize the nonlinear tolerance capability of the entire receiving link. In the meantime, under the working condition of weak received echo signals, whether the inherent noise generated inside the receiving link can overwhelm the effective energy of target echo signals is mainly determined by the overall noise figure performance of the receiving channel.

**Figure 8:**
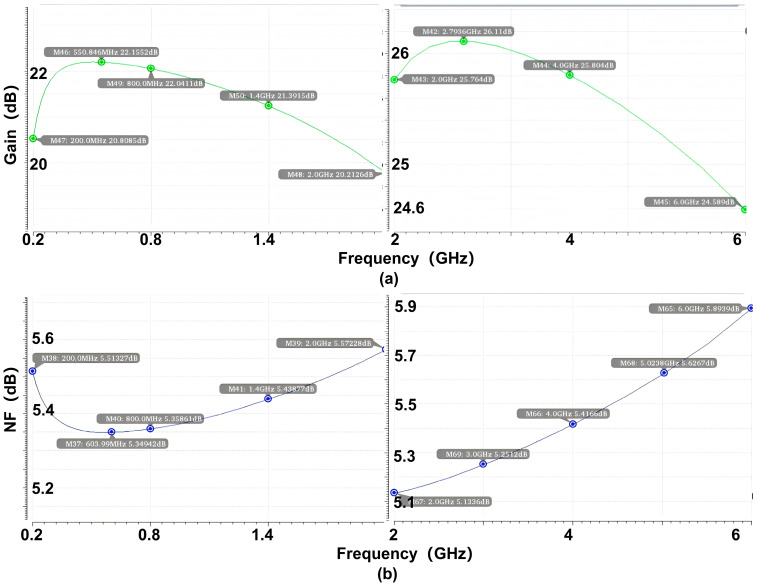
Simulation results of the receiver (**a**) gain and (**b**) NF.

**Figure 9:**
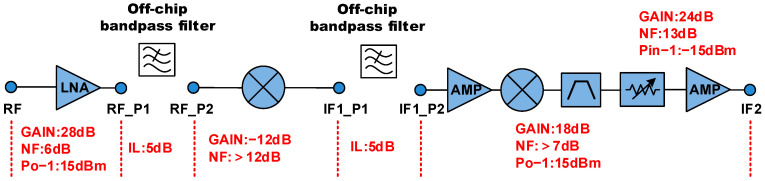
Simulation results of the receiving chain performance. LNA: low noise amplifier; AMP: amplifier; RF: radio frequency; IF: intermediate frequency.

Figure 8 presents the simulation results of the receive chain of the RF SoC. In this designed system solution, the maximum allowable input amplitude of the built-in analog-to-digital converter integrated inside the proposed wideband digital transceiving ASIC chip is set to 10 dBm, and its corresponding equivalent noise figure parameter is 35 dB. As clearly demonstrated by the systematic simulation results presented in Figure 9, the maximum achievable gain of the designed radio frequency receiving link is 24 dB, the overall system noise figure is optimized to 13 dB, and the measured input 1 dB compression point can reach −15 dBm. In addition, the analog-to-digital converter module and the first-stage low-noise amplifier arranged in the whole receiving link can enter the saturation state synchronously without generating any loss of the effective dynamic range of the analog-to-digital conversion unit. All aforementioned key performance parameters meet the comprehensive technical specification standards formulated for the receiving channel of the whole radar system.

The noise figure is measured via the Y-factor method, as expressed in the following formula:

(1) $$NF(dB)=ENR(dB)-10\mathrm{lg}(Y),\;Y=\frac{(S/N)out,on}{(S/N)out,off}$$

### 3.3. Simulation Results of Thermal Characteristics

During the formal operational process of the overall integrated system, both the radio frequency SoC and the digital transceiving ASIC chip feature a relatively compact physical layout and a small chip footprint, which inevitably leads to a relatively high internal power consumption density of such core semiconductor devices. Under such operating characteristics, reasonable and reliable heat dissipation design has become an indispensable core guarantee to ensure the long-term stable and continuous reliable operation of the whole transceiving system under various working conditions.

In view of the above thermal management demands, the proposed integrated module adopts high-performance thermally conductive gaskets to achieve tight and reliable thermal contact connection between the core high heat-dissipating devices arranged on the printed circuit board module and the protruding thermal conduction bosses reserved on the outer structural shell of the assembly. Meanwhile, dedicated liquid cooling flow channels are rationally arranged and embedded inside the inner space of the structural shell, which can efficiently and rapidly conduct and dissipate excessive heat generated by high-power operating devices in real time.

**Figure 10:**
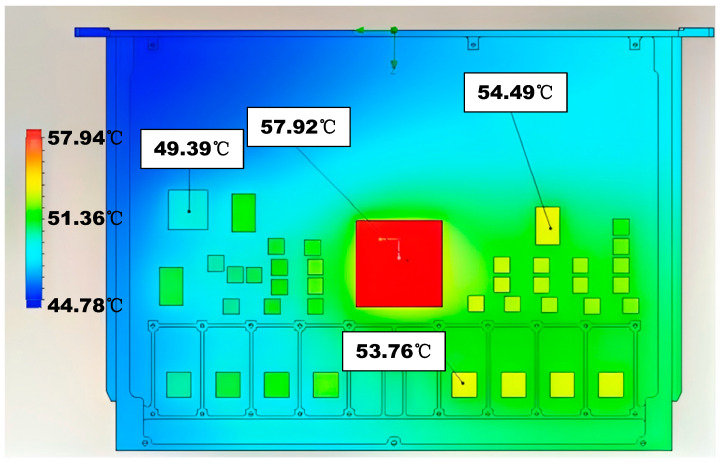
Thermal simulation results.

It can be clearly seen from the thermal simulation results presented in Figure 10 that the electronic device with the maximum internal junction temperature in the whole working system has been identified. When the external ambient temperature is maintained steadily at 25 °C, the actual internal junction temperature of this key device can reach up to 59 °C. The measured temperature stays within the allowable range and meets the derating specifications and long-term operation requirements for engineering applications.

## 4. Test and Result Analysis

**Figure 11:**
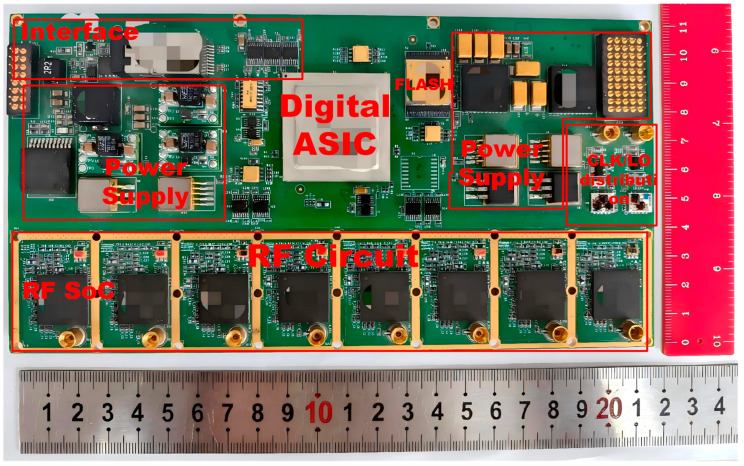
Photograph of the proposed RF transceiver module.

Figure 11 presents a physical photograph of the RF transceiver module proposed in this paper, with overall dimensions of 215 × 110 × 13.5 mm.

**Figure 12:**
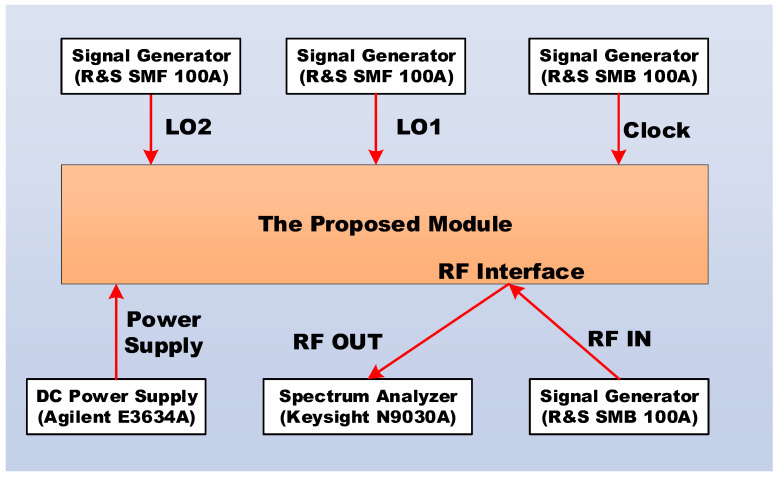
The schematic diagram of the test environment.

Figure 12 presents the test setup diagram. The E3634A DC power supply (Keysight Technologies Inc., Santa Rosa, CA, USA) provides power for the DUT. Two high-performance signal generators (SMF 100A, Rohde & Schwarz GmbH & Co. KG, Munich, Germany) supply the local oscillator signals LO1 and LO2, while one SMB 100A signal generator outputs the clock signal. For reception tests, an additional SMB 100A signal generator is connected to the RF port of the proposed module. For transmission tests, the RF port is linked to the N9030A spectrum analyzer (Keysight Technologies Inc., Santa Rosa, CA, USA). For RF cable calibration, the cable is directly connected between the signal generator and the spectrum analyzer. The measured cable loss is 2.5 dB.

**Figure 13:**
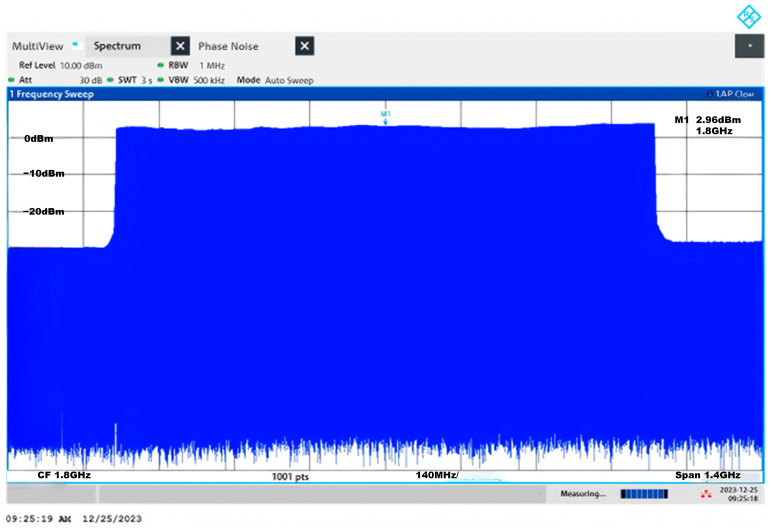
Spectrum of transmit broadband test.

As shown in Figure 13, the test results of wideband transmitting performance are presented. The wideband digital transceiving ASIC chip generates intermediate frequency signals centered at 650 MHz. These signals are further processed inside the RF SoC through filtering, frequency conversion and driving amplification. Ultimately, the system outputs radio frequency signals with a center frequency of 1.8 GHz and an RF bandwidth of 1 GHz. Compared with the original 0.2 GHz bandwidth, the bandwidth performance is improved by 5 times. After compensating for the 5 dB attenuation introduced by calibrated test cables, the in-band output power exceeds 4 dBm, and the in-band amplitude flatness is better than 4 dB. All measured performance parameters fully meet the design specifications and technical requirements for the transmitting channel of the whole system.

**Figure 14:**
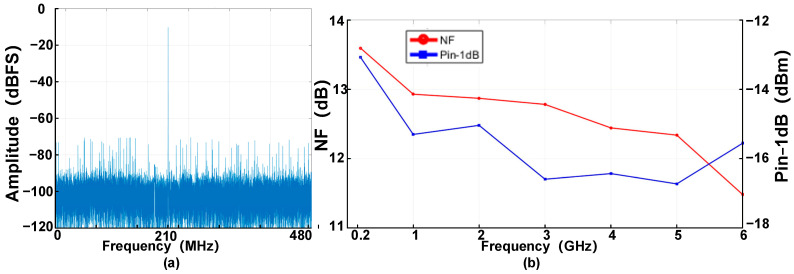
(**a**) Spectrum and (**b**) noise figure of the receiving test.

The experimental measurement results of the receiving link are presented in Figure 14. During the actual testing process, the sampling clock frequency of the analog-to-digital converter is configured to 960 MHz. After completing digital sampling processing, the intermediate frequency signal of 650 MHz is converted to a corresponding frequency component of 210 MHz in the final frequency spectrum. The amplitude of the input test signal is set to −10 dBFS, and the overall spurious suppression level of the designed system can reach more than 60 dBc. Within the full operating frequency range covering from 0.2 GHz to 6 GHz, the measured noise figure of the receiving link is consistently kept below 13.6 dB, and the input 1 dB compression point is higher than −16.7 dBm across the entire frequency band. All the actual measured performance indicators are basically consistent with the preset design targets and previous simulation prediction results, which verifies the rationality and practicability of the proposed receiving link design scheme.

The measured power consumption test results are presented in the Table 1 and Table 2. The total power consumption of the eight-receiver and eight-transmitter digital transceiving system designed in this work reaches 40.9 W. Among the overall power consumption of the whole system, the power consumption proportion occupied by the frequency conversion SoC is 21.5%, while that of the digital ASIC chip accounts for 53.1%, and the overall power supply efficiency of the complete system is calculated to be 82%. For comparative analysis, the traditional eight-receiver and eight-transmitter digital transceiving system constructed based on state-of-the-art commercial process chips, which adopts a discrete hardware architecture composed of independent radio frequency chips, high-speed ADC and DAC devices, as well as dedicated FPGA control chips, possesses a total power consumption as high as 91.1 W. Through comprehensive data comparison and quantitative analysis, it can be clearly concluded that the total power consumption of the ASIC-centered system architecture proposed in this paper is reduced by 55.1% in contrast with the conventional structural scheme, which verifies the obvious power-saving advantage of the proposed fully ASIC-based architecture in practical engineering applications.

In size, the eight-channel module with mixing link achieves a 53.1% area reduction versus our conventional eight-channel hardware board, with an obvious miniaturization effect.

**Figure 15:**
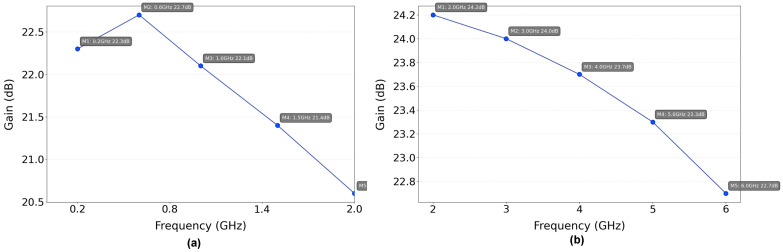
Receiving gain measurement results for (**a**) 0.2–2 GHz band and (**b**) 2–6 GHz band.

**Figure 16:**
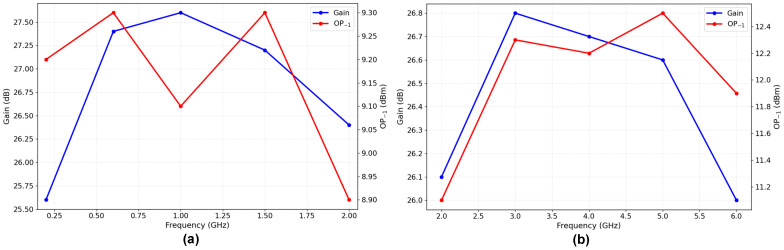
Transmit gain and OP − 1 measurement results for (**a**) the 0.2–2 GHz band and (**b**) the 2–6 GHz band. OP − 1: Output 1 dB compression point.

Figure 15 and Figure 16 present the measured results of receiving gain, transmit gain, and transmit OP − 1 (Output 1 dB compression point), respectively.

It should be emphasized that only module-level validation is completed in this paper, and the overall validation of the phased-array system will be conducted in follow-up work.

## 5. Conclusions

Addressing the longstanding pain points of traditional RF transceiver modules—poor inter-platform universality, large physical size, and high power consumption—this paper has proposed and demonstrated an ultra-compact, universal transceiver module based on an ASIC-centered architecture. By synergistically integrating a broadband RF SoC and a reconfigurable digital SoC, the design achieves complete transceiver chain integration, and resolves the insufficient universality of traditional tailored solutions.

Test results for the eight-channel prototype confirm a 53.1% footprint reduction and a total power consumption of only 40.9 W, representing a 55.1% decrease compared with traditional discrete schemes. All critical RF performance metrics meet the rigorous demands of advanced SAR and phased-array systems. The approach thus provides an effective, low-cost, and rapid-development technical pathway for transceiver modules across radar and communication domains and carries significant engineering application value for next-generation digital arrays.

Future work will focus on further optimizing the chip fabrication process and the on-chip architecture to push the integration level and instantaneous bandwidth into millimeter-wave frequencies. We also plan to scale the number of channels and explore three-dimensional heterogeneous integration to address the requirements of large-scale, ultra-dense phased-array antennas for future sensing and communication networks.

## Figures and Tables

**Table 1:** Performance comparison.

Item	Proposed Design	Original Design [28]
Size (mm × mm × mm)	215 × 110 × 13.5	280 × 180 × 13.5 ^a^
Power Consumption (W)	40.9	91.1 ^a^
Transmit Bandwidth (GHz)	1	1.2 ^b^
Flatness (dB)	4	2.07 ^b^
Spurious Suppression (dBc)	60	58.1 ^b^

^a^ Measured using our conventional 8-channel hardware board. ^b^ Extracted from the cited reference [28].

**Table 2:** Detailed power consumption comparison.

Component of the Proposed Design	Component Power Consumption of the Proposed Design	Component of the Original Design	Component Power Consumption of the Original Design
RF SoC	7.2 W	RF SiP	17 W
Digital ASIC	17.8 W	ADC (CX8242)	9.6 W
LO distribution	2 W	DAC (CX8242)	9.6 W
CLK distribution	2 W	FPGA (JFM7V690T)	30 W
Interface	4.5 W	LO distribution	2 W
Power Supply Efficiency	82%	CLK distribution	2 W
Total	40.9 W	Optical interface	4.5 W
		Power Supply Efficiency	82%
		Total	91.1 W

## Data Availability

The original contributions presented in this study are included in the article. Further inquiries can be directed to the corresponding author.

## Abbreviations

The following abbreviations are used in this manuscript:

SAR	Synthetic Aperture Radar
T/R	Transmit/Receive
RF	Radio frequency
IF	Intermediate frequency
ASIC	Application-specific integrated circuit
SoC	System-on-chip
DDC	Digital down converters
DUC	Digital up converters
LO	Local oscillator
ADC	Analog-to-Digital Converter
DAC	Digital-to-Analog Converter
FPGA	Field Programmable Gate Array
PCB	Printed Circuit Board
LTCC	Low Temperature Co-fired Ceramic
MEMS	Micro-Electro-Mechanical Systems
SAW	Surface Acoustic Wave
DC-DC	Direct Current to Direct Current
LDO	Low Dropout Regulator
PLL	Phase-Locked Loop
PA	Power Amplifier
LNA	Low Noise Amplifier
SerDes	Serializer/Deserializer
I/O	Input/Output
CLK	Clock
OP-1	Output 1 dB compression point

## References

[1] D’Errico M. (2012). Distributed Space Missions for Earth System Monitoring.

[2] Wang R., Liu K., Liu D., Ou N., Yue H., Chen Y., Yu W., Liang D., Cai Y. (2025). LuTan-1: An innovative L-band spaceborne bistatic interferometric synthetic aperture radar mission. IEEE Geosci. Remote Sens. Mag..

[3] Liang D., Zhang H., Liu K., Liu D., Wang R. (2022). Phase Synchronization Techniques for Bistatic and Multistatic Synthetic Aperture Radar: Accounting for frequency offset. IEEE Geosci. Remote Sens. Mag..

[4] Farquharson G., Castelletti D., Stringham C., Ryu J., Yague-Martinez N., Goncharenko Y., De S., Cazcarra-Bes V. (2025). Capella Space: The first seven years. IEEE Geosci. Remote Sens. Mag..

[5] Wang R., Deng Y. (2018). Bistatic SAR System and Signal Processing Technology.

[6] Moreira A., Prats-Iraola P., Younis M., Krieger G., Hajnsek I., Papathanassiou K.P. (2013). A tutorial on synthetic aperture radar. IEEE Geosci. Remote Sens. Mag..

[7] Deng Y., Yu W., Wang P., Xiao D., Wang W., Liu K., Zhang H. (2024). The High-Resolution Synthetic Aperture Radar System and Signal Processing Techniques: Current progress and future prospects. IEEE Geosci. Remote Sens. Mag..

[8] Li B., Nan Y.J., Li J.F., Lu P.P., Wang R., Liang D. (2024). A Novel Nonlinear Frequency Scanning SAR Imaging Mode. IEEE Trans. Geosci. Remote Sens..

[9] Zhou T., Zhang X.Y., Chen L.M. (2025). A Novel Phase Synchronization Method for Spaceborne Multistatic SAR. IEEE Trans. Geosci. Remote Sens..

[10] Nazar M.L., Khan A., Shah S.A. (2025). Design and Implementation of a Low Cost, Low Profile, Multipurpose FMCW RADAR Transceiver. Proceedings 2025 IEEE International Conference on Microwave and Millimeter Wave Technology (ICMMT).

[11] Kanfade A., Kumar V., T V. (2025). A Modular, Scalable and High Performance Design for Multi-Channel Wideband Transceivers. 2025 IEEE Microwaves, Antennas, and Propagation Conference (MAPCON), Kochi, India.

[12] Wang S. (2025). Design and Implementation of S-band Dual-channel RF Direct Digital Transceiver. 2025 13th International Conference on Information and Communication Networks (ICICN), Beijing, China.

[13] Mangal J., Choudhary A., Darak S., Ram S.S. (2026). High Resolution Range Estimation Using Millimeter Wave Integrated Sensing and Communication on RFSoC. 2026 18th International Conference on COMmunication Systems and NETworks (COMSNETS), Bengaluru, India.

[14] Chen H.W., Li J.B., Wang Z.H. (2026). Joint Design of Low Sidelobe Radar Waveform and Filter with Hardware Platform Verification. IEEE Trans. Aerosp. Electron. Syst..

[15] Chen Y., Cai Y., Li B., Li J., Nan Y., Liang D., Lu P., Wang R. (2024). Narrowband RFI Suppression for Phase Synchronization of BiSAR Based on Robust Principal Component Analysis. EUSAR 2024; 15th European Conference on Synthetic Aperture Radar, Munich, Germany.

[16] Suematsu N., Furuichi T., Tsukamoto S., Morino Y., Hirai A. (2025). Q/V-band DBF array antenna and direct digital RF transceivers for future LEO constellation satellites. 2025 Asia-Pacific Microwave Conference (APMC).

[17] Yang Z., Xia X., Liu Y., Wen G., Zhang W.E., Guo L. (2024). LPST-Det: Local-perception-enhanced Swin Transformer for SAR ship detection. Remote Sens..

[18] Qiu T., Wang P., Wang Y., He T., Chen J. (2026). A novel in-orbit approach for spaceborne SAR absolute radiometric calibration using a small calibration satellite. Remote Sens..

[19] Wei M., Jiang G., Zou L., Wen X., Li Z. (2026). Geothermal resource exploration using multi-temporal infrared remote sensing data based on annual temperature variation model. Remote Sens..

[20] Lei W., Zhang Y., Xie H., Chen Z., Chen Z., Li W. (2025). A fully digital transmitting-receiving platform for MIMO radar waveform diversity experiment. IEICE Trans. Inf. Syst..

[21] Xie J., Fu Z. (2025). A 72-Channel RF transceiver with High Flatness for Massive MIMO Channel Emulator. 2025 18th IEEE United Conference on Millimeter Waves and Terahertz Technologies (UCMMT), Nanjing, China.

[22] Singh N.K. (2024). Multi-board Synchronization of n-channel Direct RF Digital Transceiver for Active Phased Array Radar using JESD204 Interface. 2024 IEEE 9th International Conference for Convergence in Technology (I2CT), Pune, India.

[23] Marsic V., Faramehr S., Fleming J., Ball P., Ou S., Igic P. (2023). Buried RF sensors for smart road infrastructure: Empirical communication range testing, propagation by line of sight, diffraction and reflection model and technology comparison for 868 MHz–2.4 GHz. Sensors.

[24] Jin R., Cheng J., Wang W., Zhang H., Zhang J. (2024). Attribute feature perturbation-based augmentation of SAR target data. Sensors.

[25] Li X., He P., Song J., Wang Z. (2025). Receiver location optimization for heterogeneous S-band marine transmitters in passive multistatic radar networks via NSGA-II. Sensors.

[26] Badoni D., Ammendola R., Bocci V., Chiodi G., Iacoangeli F., Pasta S., Rebustini G., Recchia L. (2026). Analog front-end ASIC for compact silicon photomultiplier sensor interfaces in mixed-signal systems. Sensors.

[27] Liang Z., Guo W., Wang C., Liu P., Yang S. (2026). Development of channelized K/V band Dicke microwave radiometer based on SDR. Sensors.

[28] Piao J.M., Yu C.P., Liu Y.A. (2025). 1.2GHz Wideband Radio Frequency Transceiver System. 2025 IEEE 8th International Symposium on Electromagnetic Compatibility (ISEMC).

[29] Fang T., Deng Y., Liang D., Zhang L., Zhang H., Fan H., Yu W. (2022). Multichannel Sliding Spotlight SAR Imaging: First Result of GF-3 Satellite. IEEE Trans. Geosci. Remote Sens..

[30] Liang D., Wang R., Deng Y., Fan H., Zhang H., Zhang L., Wang W., Zhou Y. (2019). A Channel Calibration Method Based on Weighted Back projection Algorithm for Multichannel SAR Imaging. IEEE Geosci. Remote Sens. Lett..

